# A Curious Presentation of a Human Papillomavirus (HPV)-Driven Pelvic Squamous Cell Carcinoma of Unknown Primary: A Case Report

**DOI:** 10.7759/cureus.82134

**Published:** 2025-04-12

**Authors:** Georgia E Dau, Alexandra K Brennan, Nathan J Teschan, Jolyn S Taylor, Stuart S Winkler

**Affiliations:** 1 Obstetrics and Gynecology, Brooke Army Medical Center, San Antonio, USA; 2 Radiation Oncology, Brooke Army Medical Center, San Antonio, USA; 3 Pathology, Brooke Army Medical Center, San Antonio, USA; 4 Gynecologic Oncology and Reproductive Medicine, The University of Texas MD Anderson Cancer Center, Houston, USA; 5 Gynecologic Oncology, Brooke Army Medical Center, San Antonio, USA

**Keywords:** carcinoma of unknown primary (cup), hpv-associated malignancy, human papillomavirus (hpv), occult carcinoma, scc of unknown primary, squamous cell carcinoma (scc), squamous cell rectal carcinoma

## Abstract

Cancers of unknown primary (CUP) are rare malignancies among invasive cancers and are comprised of a variety of subtypes. In the pelvis, occult squamous cell carcinoma (SCC) usually arises from the vulva, vagina, cervix, or anus. We present the case of a 55-year-old patient with pelvic pain. Magnetic resonance imaging showed a presacral and posterior lower uterine soft tissue mass measuring 6.7 x 5.0 x 6.2 cm with regional lymph node involvement. An exam under anesthesia and colonoscopy did not detect cervical or anal involvement. A biopsy of the mass confirmed poorly differentiated SCC, positive p16 staining on immunohistochemistry, and human papillomavirus (HPV+) RNA in-situ hybridization. The findings were most consistent with an atypical presentation of locally advanced cervical cancer. The patient received chemoradiation with volumetric modulated arc therapy. After four weeks of treatment with some tumor response, she received stereotactic body radiotherapy (SBRT) as the mass was not accessible for brachytherapy. The patient ultimately had a partial response with the shrinking tumor separating from the cervix. She was re-evaluated by a multidisciplinary team, and based on response and imaging, the tumor was most likely determined to originate from the anus. Colorectal surgery assumed care for definitive abdominal perineal resection. This case demonstrates the importance of continuous evaluation of the site of disease in patients with CUP and the avoidance of anchoring on a definitive diagnosis. Identification of the primary site may be impossible initially, but willingness to alter treatment as new information presents is essential to improve patient outcomes.

## Introduction

Cancers of unknown primary (CUP) are rare malignancies in which the primary site cannot be identified during initial evaluation. CUPs consist of approximately 2% of all invasive cancers [1]. CUP subtypes include adenocarcinoma, undifferentiated carcinoma, squamous cell carcinoma (SCC), poorly differentiated malignant neoplasms, and neuroendocrine tumors. During the initial evaluation, which includes tissue biopsy and pathologic evaluation, a primary site will be identified in only 30% of patients [1]. Within these CUP subtypes, SCC makes up just 5% of cases. These carcinomas are aggressive and characterized by early spread, absence of clinical symptoms, and resistance to treatment [2]. Over half of patients will have multiple sites of disease at diagnosis.

Human papillomavirus (HPV) is a double-stranded DNA virus that commonly infects the epithelial sites of sexually active individuals by integrating its DNA into the host genome. The detection of HPV in tissue specimens is aided by polymerase chain reaction (PCR), immunohistochemistry (IHC), and in-situ hybridization (ISH). The IHC evaluation of tumor suppressor protein p16 helps distinguish HPV-related SCC [3]. HPV commonly infects squamous cells of the anogenital tract, leading to viral integration into the host cells. The production of the E6 and E7 proteins causes degradation of the tumor suppressor pathways involving p53 and Rb (retinoblastoma protein), leading to the characteristic p16 overexpression. However, non-HPV-related carcinomas can also overexpress p16 from Rb inactivation through independent mechanisms [3]. In these cases, ISH is a highly sensitive method of detecting HPV [4]. We report a case of a poorly differentiated SCC of unknown origin in the presacral region.

## Case presentation

**Figure 1:**
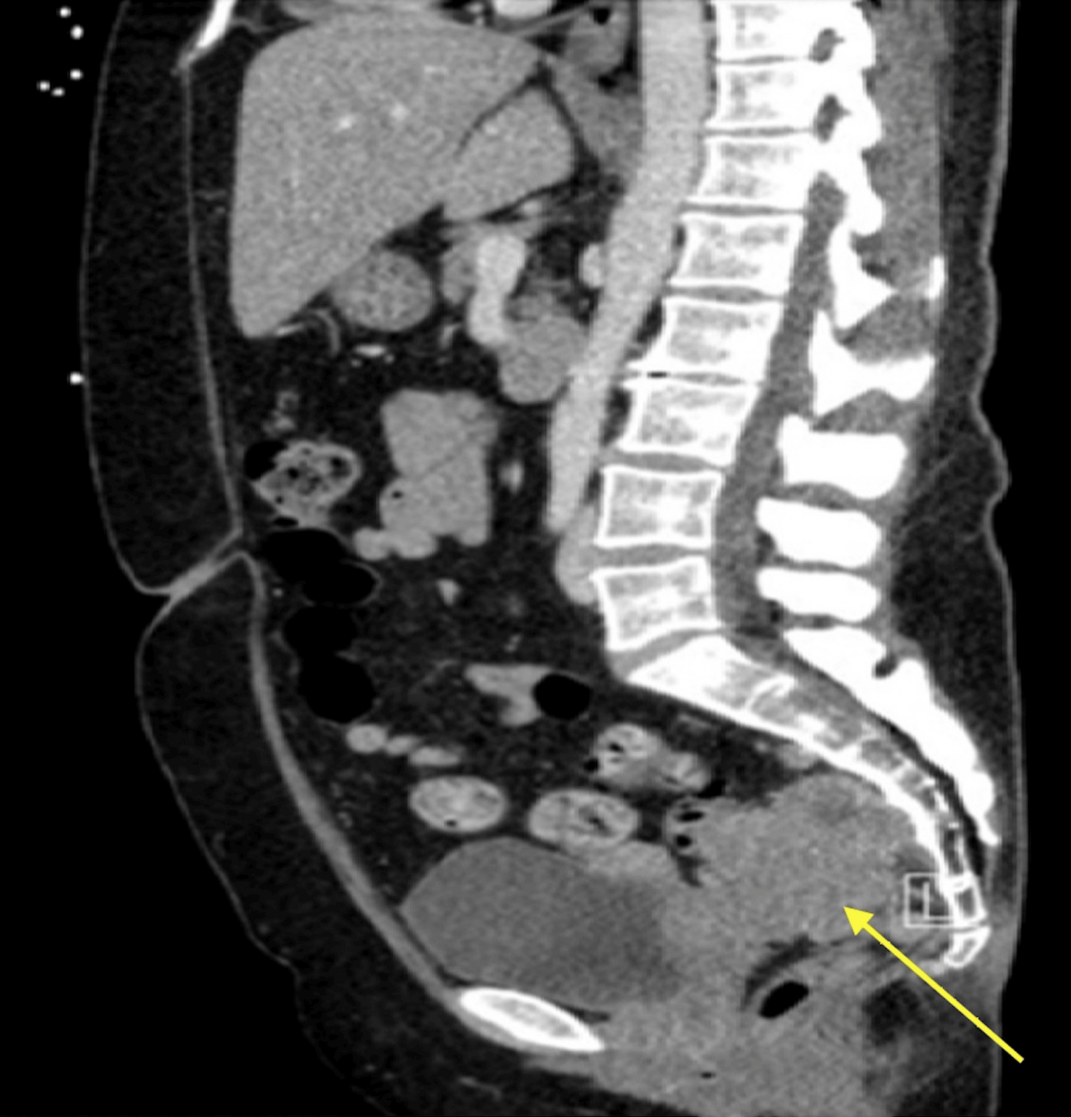
Sagittal CT showing heterogeneously enhancing presacral, posterior lower uterine soft tissue mass (yellow arrow) measuring 6.7 x 5.0 x 6.2 cm.

Our patient is a postmenopausal 55-year-old woman who presented to her primary care provider with lower back pain. Her medical history included anxiety, depression, and fibromyalgia. She had no known history of cervical dysplasia but had limited screening over the previous 15 years. Six months after the initial presentation without improvement in lower back pain, a computed tomography (CT) was performed and showed a large heterogeneously enhancing presacral, posterior lower uterine soft tissue mass measuring 6.7 x 5.0 x 6.2 cm (Figure 1). The mass abutted and displaced the superior aspect of the rectum to the left. There were separate round enhancing presacral soft tissue masses along the posterior inferior aspect of the dominant mass measuring 1.3 cm with few presacral lymph nodes measuring up to 1 cm.

**Figure 2:**
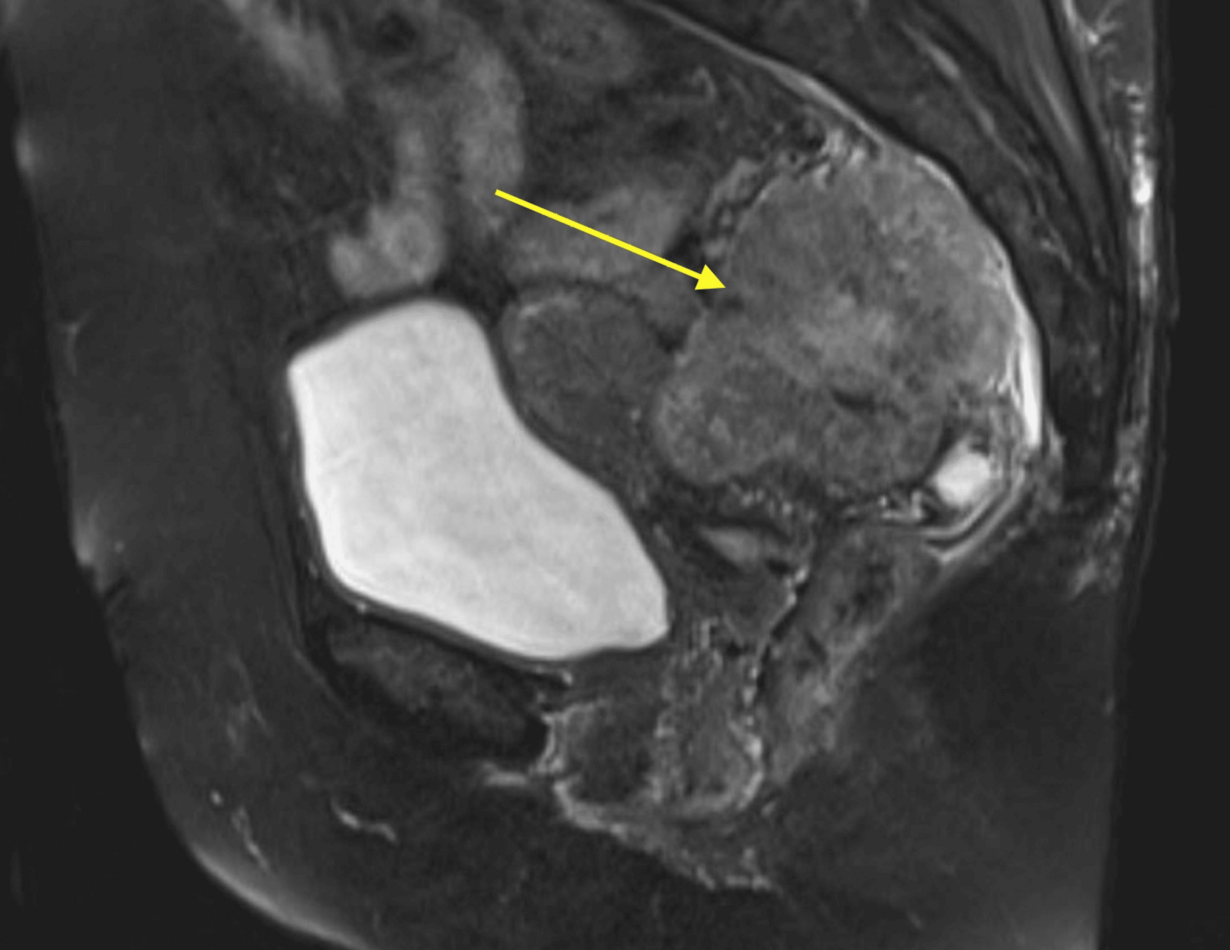
Sagittal MRI demonstrating dominant T2 pelvic mass (yellow arrow) with effect on surrounding structures. MRI: magnetic resonance imaging

A magnetic resonance imaging (MRI) study was performed, which confirmed a dominant tumor with a mass effect on adjacent pelvic structures, including the uterus and rectum, with marked diffusion restrictions (Figure 2). There was no clear delineation or separation from the uterus.

**Figure 3:**
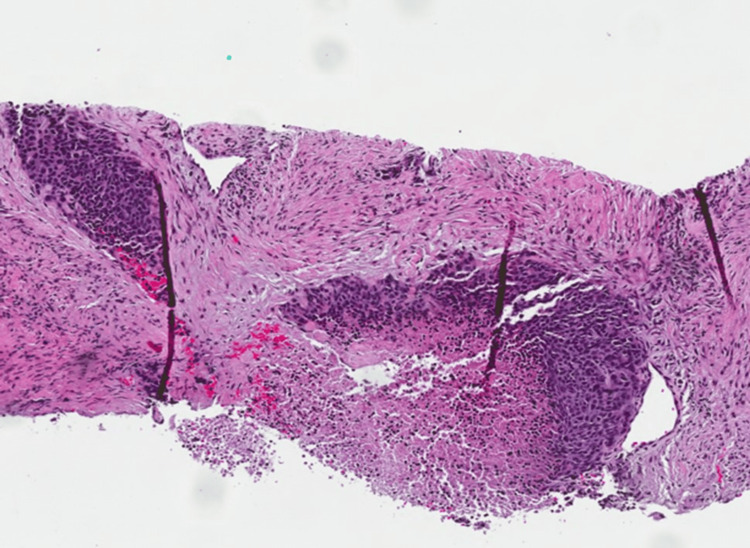
H&E stain demonstrating invasive groups of epithelioid, cohesive cells with high nuclear-to-cytoplasmic ratios, marked nuclear pleomorphism, and atypia with tumor cell necrosis. H&E: hematoxylin and eosin

**Figure 4:**
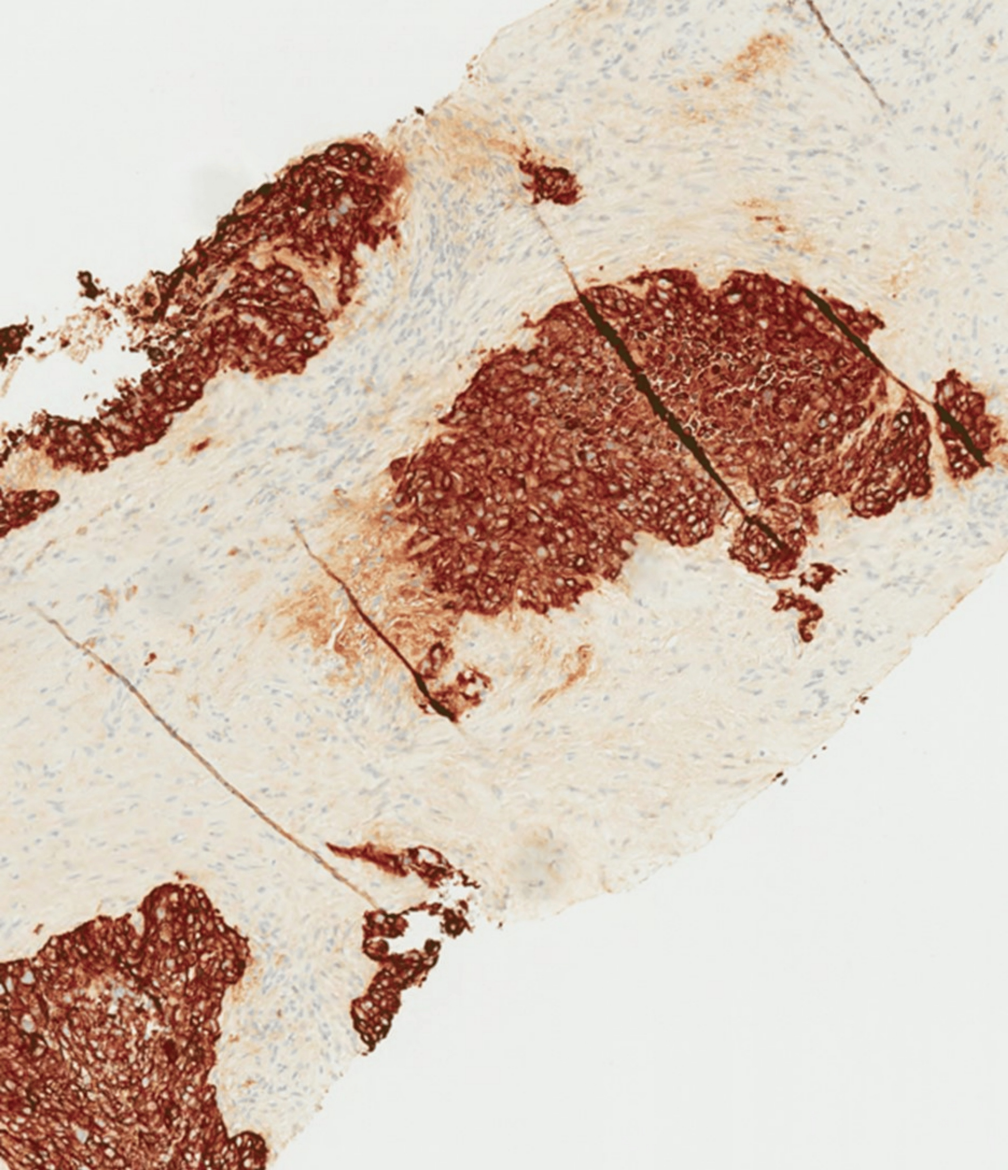
AE1/AE3 demonstrating membranous staining throughout the tumor cells, which supports the diagnosis of carcinoma.

**Figure 5:**
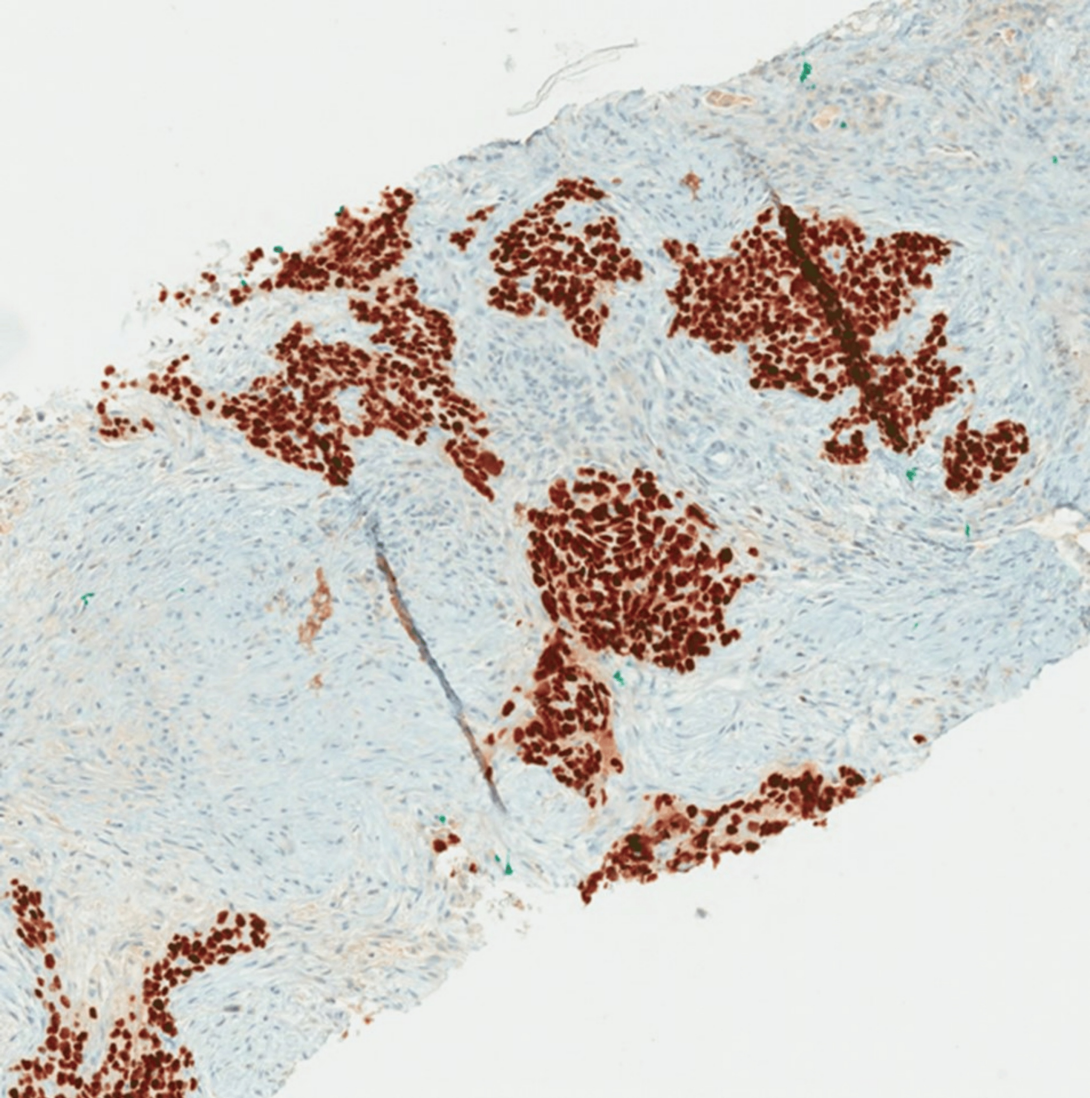
P40 strongly stains the nuclei of tumor cells throughout, which is supportive of squamous differentiation.

The presacral mass was biopsied by interventional radiology (IR) and found to be poorly differentiated SC. The tumor was predominantly fibrotic tissue with few nests of poorly differentiated epithelioid cells (Figure 3). IHC staining was positive for AE1/AE3 pancytokeratin, p40, and p16 and negative for CK7, CK20, SATB2, and PAX-2 (Figures 4, 5). An HPV RNA probe analysis identified HPV (unspecified 16/18 positive). PD-L1 was unable to be tested due to insufficient tumor tissue on the initial biopsy.

**Figure 6:**
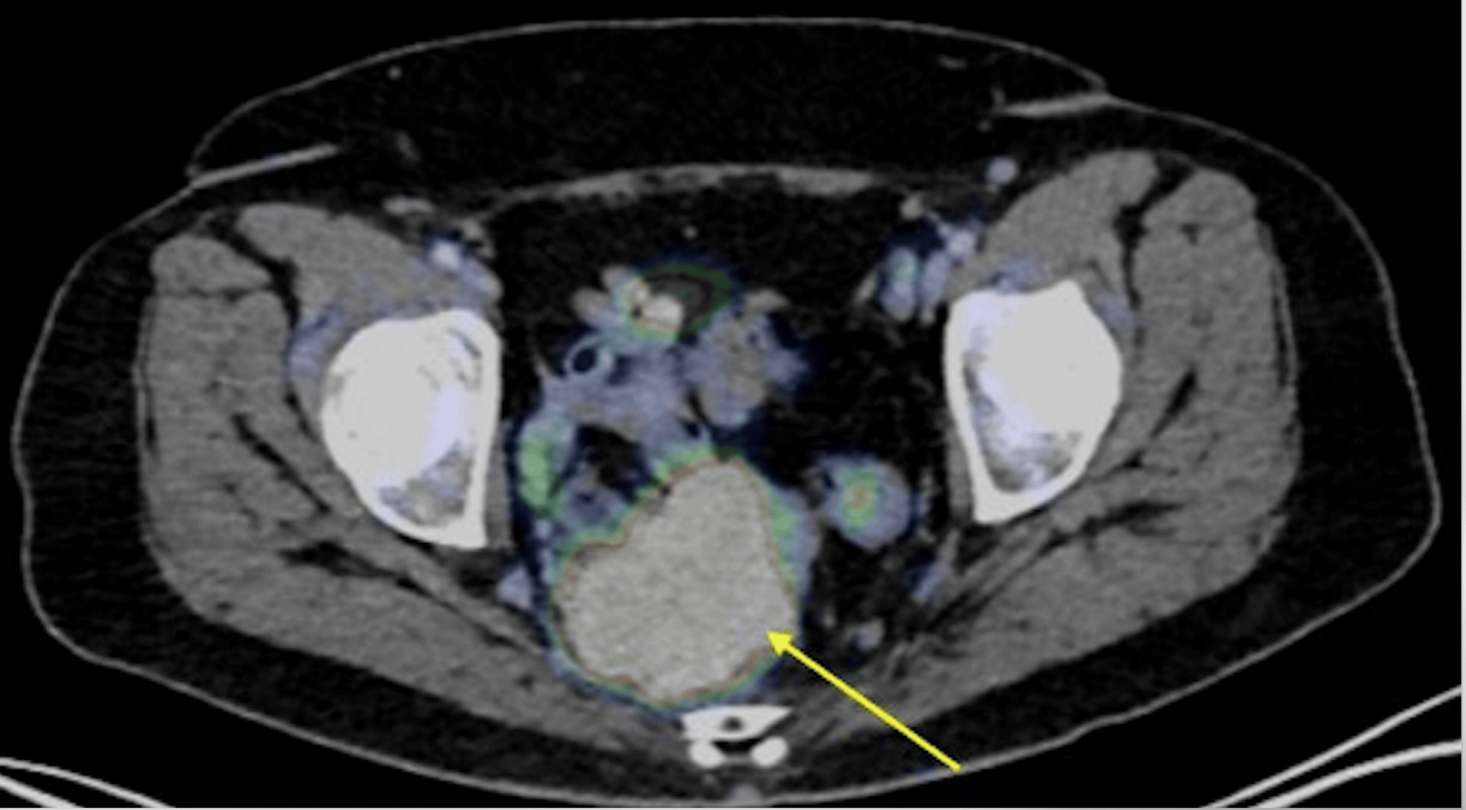
Axial fused PET/CT image demonstrating a large presacral mass (yellow arrow) with concern for serosal bowel invasion. PET/CT: positron emission tomography/computed tomography

Shortly after diagnosis, she was admitted to the hospital for worsening abdominal and back pain. Subsequently, she underwent an exam under anesthesia (EUA), notable for a normal-appearing vaginal and cervical mucosa without lesions. The posterior cervical lip appeared flush with the posterior cervicovaginal junction. The Pap smear results indicate atypical squamous cells of undetermined significance (ASCUS) and HPV negative. A bimanual exam revealed a bulbous, irregular posterior cervico-uterine mass slightly displaced to the right and indistinguishable from the posterior uterus and cervix. The mass was relatively mobile, not fixed to the sidewall or sacrum, and without obvious parametrial involvement. No masses were detected on the rectal exam. The uterus sounded to 8.5 cm. Positron emission tomography (PET) confirmed an intense hypermetabolic presacral mass without a clear site of origin that abuts the posterior cervix and upper rectum with high concern for serosal invasion of the bowel. Multiple pathologic regional presacral pelvic nodes were noted (Figure 6).

Flexible sigmoidoscopy showed rectal erythema on the posterior wall with focal white plaques and large external and internal hemorrhoids. The rectum biopsy revealed erosions and reactive epithelial changes without dysplasia or malignancy, which was confirmed with negative p16. The anal biopsy was benign and negative for p16.

**Figure 7:**
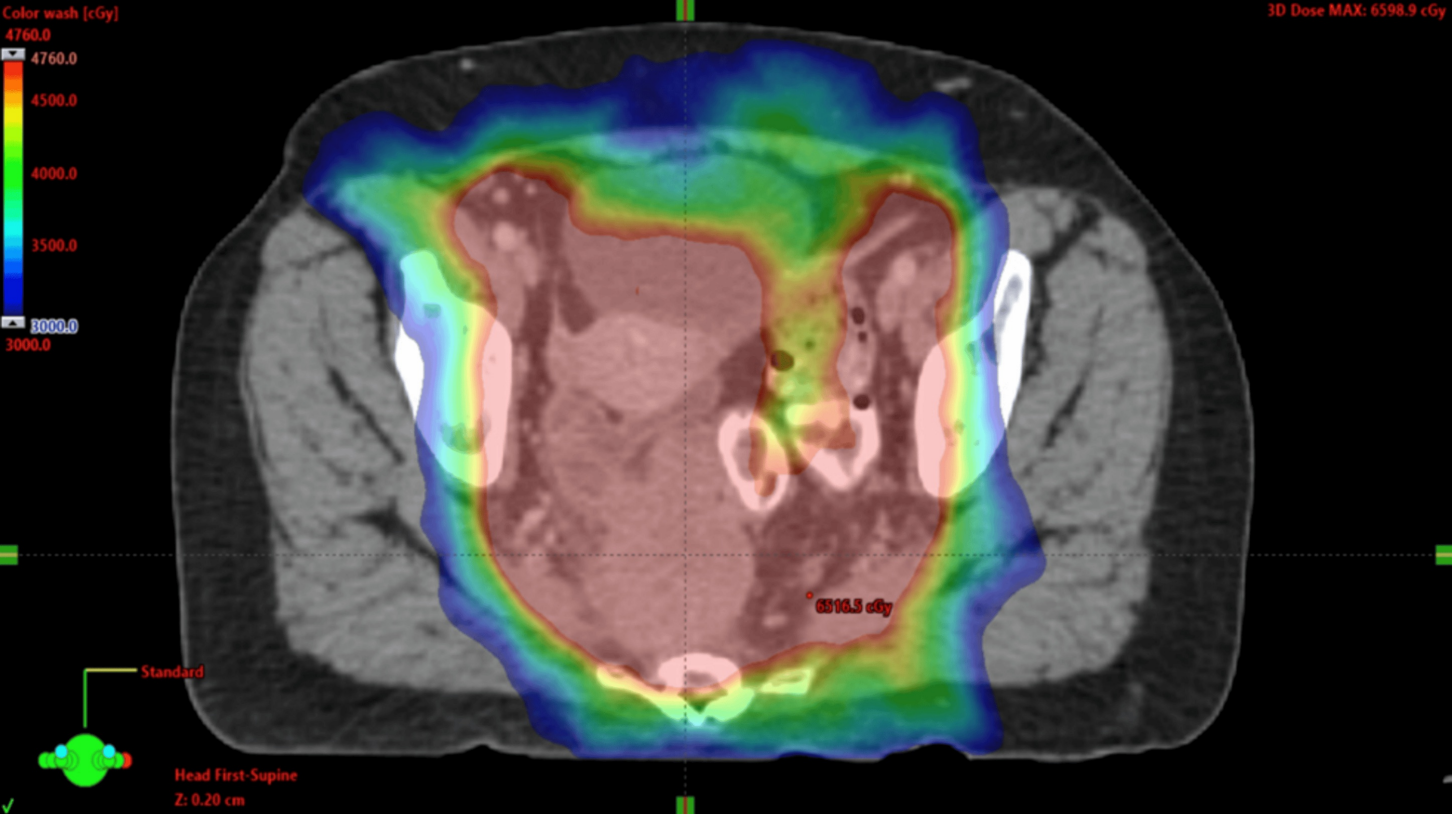
Axial PET with RT planning overlay demonstrating dose (as color wash) from 30 Gy (in blue) to 47.6 Gy (red), which is the prescription dose to the pelvis, primary, and uterus. RT: radiation tomography; PET: positron emission tomography

**Figure 8:**
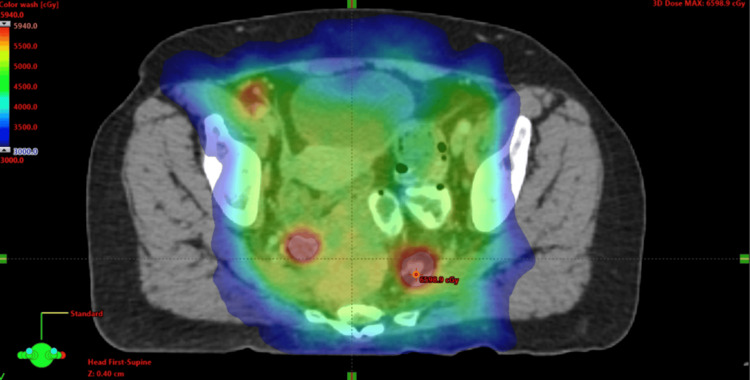
Axial PET with RT planning overlay demonstrating dose (as color wash) from 30 Gy (in blue) to 59.4 Gy (red), which is the prescription dose to the PET-positive lymph nodes. RT: radiation tomography; PET: positron emission tomography

The patient was presented at a multidisciplinary tumor board that included radiation oncology and colorectal surgery. Based on the pathology and lack of anal lesion, the tumor board recommended treatment for a locally advanced cervical cancer. She started chemoradiation with cisplatin as per the standard guidelines for the treatment of presumed cervical cancer [5]. Her initial radiation therapy involved a volumetric modulated arc therapy (VMAT) plan that included comprehensive coverage of her pelvic lymph nodes and primary tumor with a simultaneous integrated boost (SIB) to the PET-positive lymph nodes consistent with EMBRACE II [6]. Her dose regimen included 47.6 Gy to the pelvis and primary regions (Figure 7), with the concurrent boost to the nodes receiving up to 59.4 Gy over 28 fractions (Figure 8). She was treated with a comfortably full bladder, and image guidance was performed daily with a cone-beam CT (Figures 7, 8).

She underwent an interim MRI after approximately four weeks of chemoradiation therapy with a less-than-anticipated response to her primary disease. Given the location of her presumed possible primary within the pelvis and a slower-than-anticipated response, she was referred to a tertiary referral center for recommendations for treatment modalities. She was recommended to complete definitive chemoradiation with adjuvant stereotactic body radiotherapy (SBRT).

**Figure 9:**
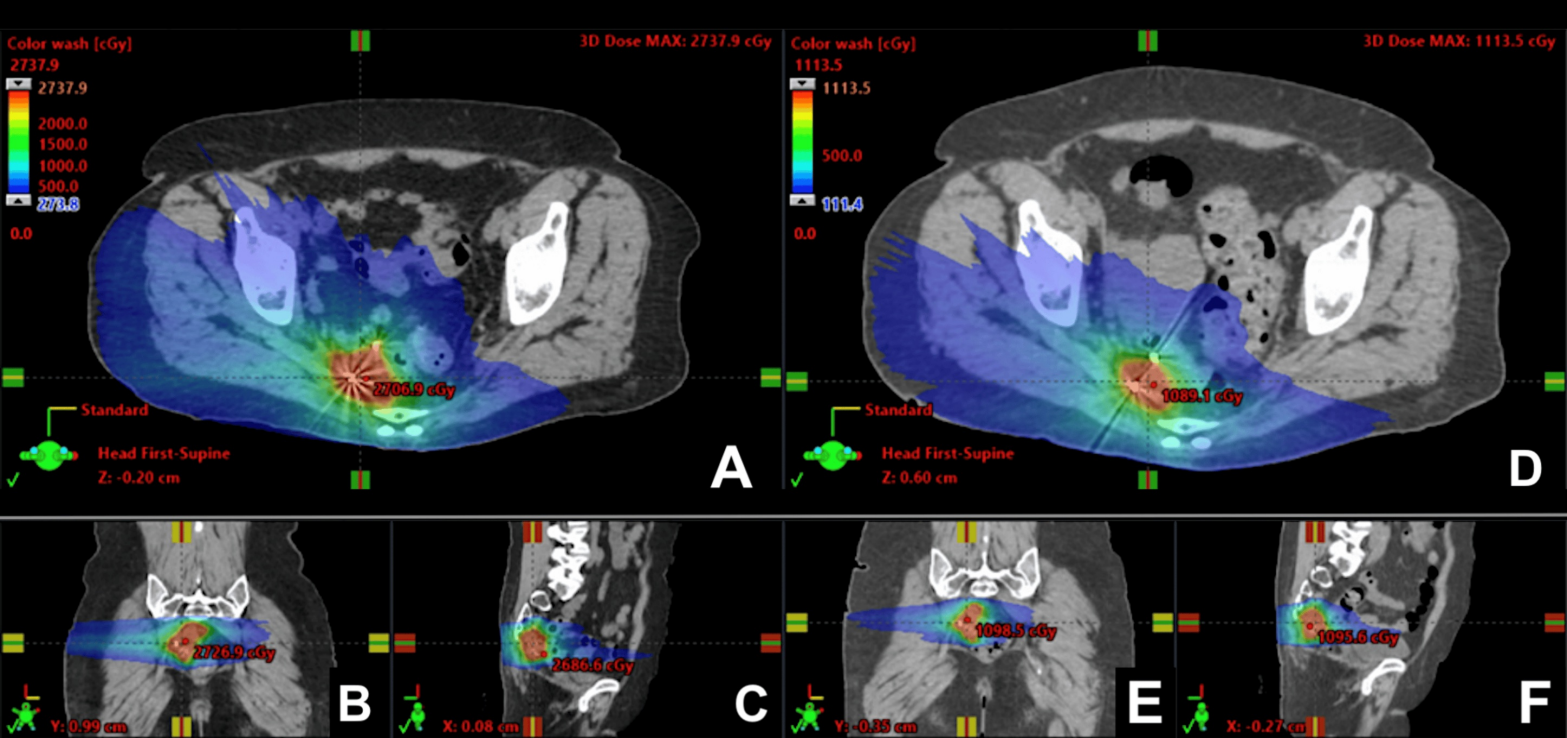
Two composite plans (A–C: F05-07 and D–F: F08-10) featuring axial (A, D), sagittal (C, F), and coronal (B, E) views of the SBRT plan with doses ranging from 2.7 Gy (blue) to 27.38 Gy (red) on the left and from 1.11 Gy (blue) to 11.13 Gy (red) on the right. The left image shows the initial SBRT plan developed. Due to shrinkage in the patient's primary, three fractions of this plan were delivered before the second plan was developed. She, therefore, received two fractions of the plan demonstrated in the right image. SBRT: stereotactic body radiotherapy

She completed five fractions of twice-weekly SBRT at 25 Gy. During SBRT, the patient's primary tumor started shrinking. She subsequently underwent an adaptive re-plan of her treatment. Her final EQD2 D2cc calculations of her organs at risk (OAR) included a bladder dose of 53.03 Gy, a small bowel dose of 62.47 Gy, a rectal dose of 65.92 Gy, a sigmoid dose of 63.41 Gy, and the final composite EQD2 D95 of her CTV (clinical target volume) was 79.68 Gy [7]. This plan is demonstrated in Figure 9.

**Figure 10:**
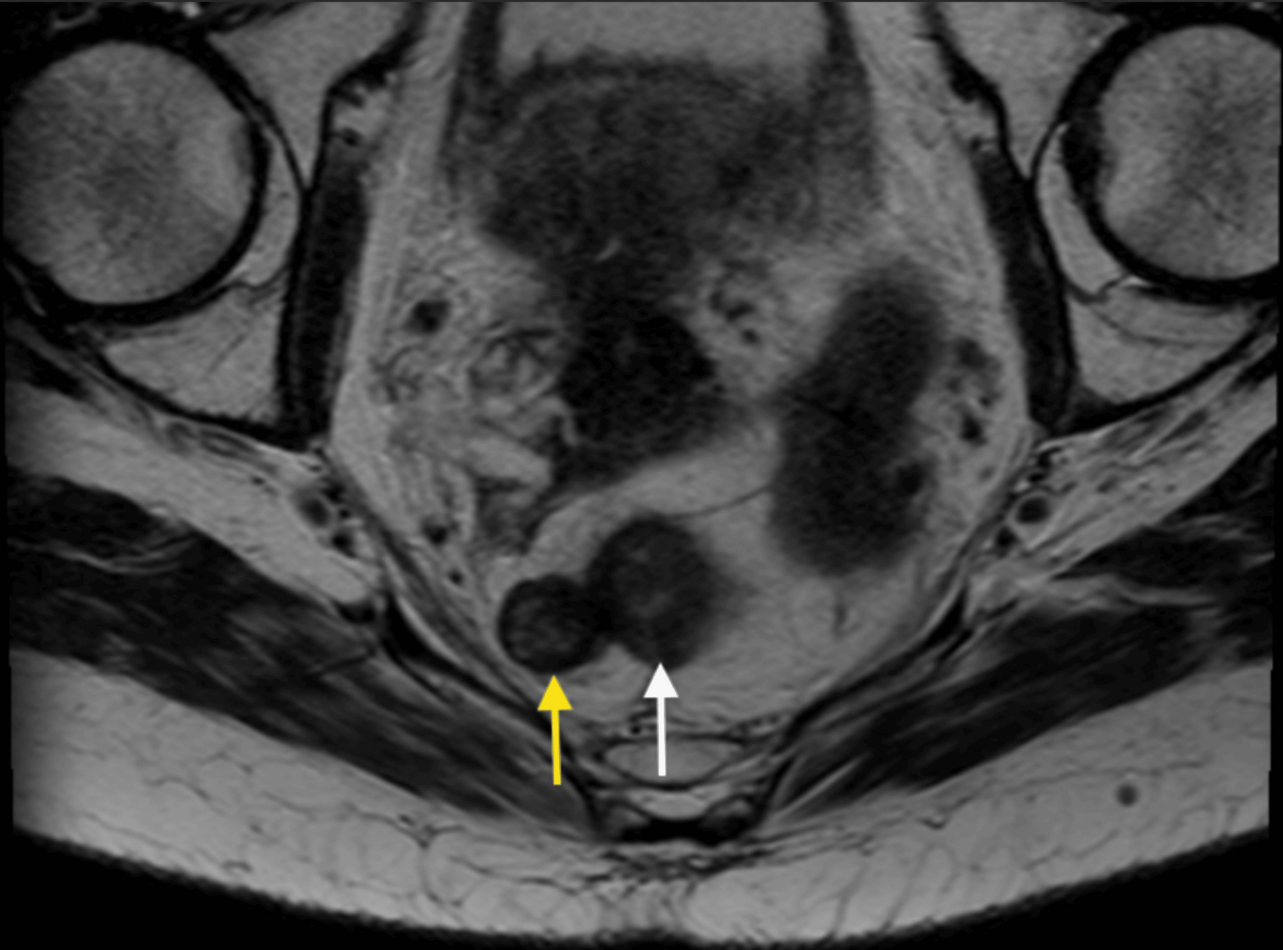
Axial MRI demonstrating a right perirectal T2 heterogeneous mass (yellow arrow) measuring 1.6 x 1.5 cm with T2 hypointense signal and T2 intermediate signal intensity with mild diffusion restriction and heterogeneous enhancement. The mass involves the right lateral rectal wall (white arrow). MRI: magnetic resonance imaging

**Figure 11:**
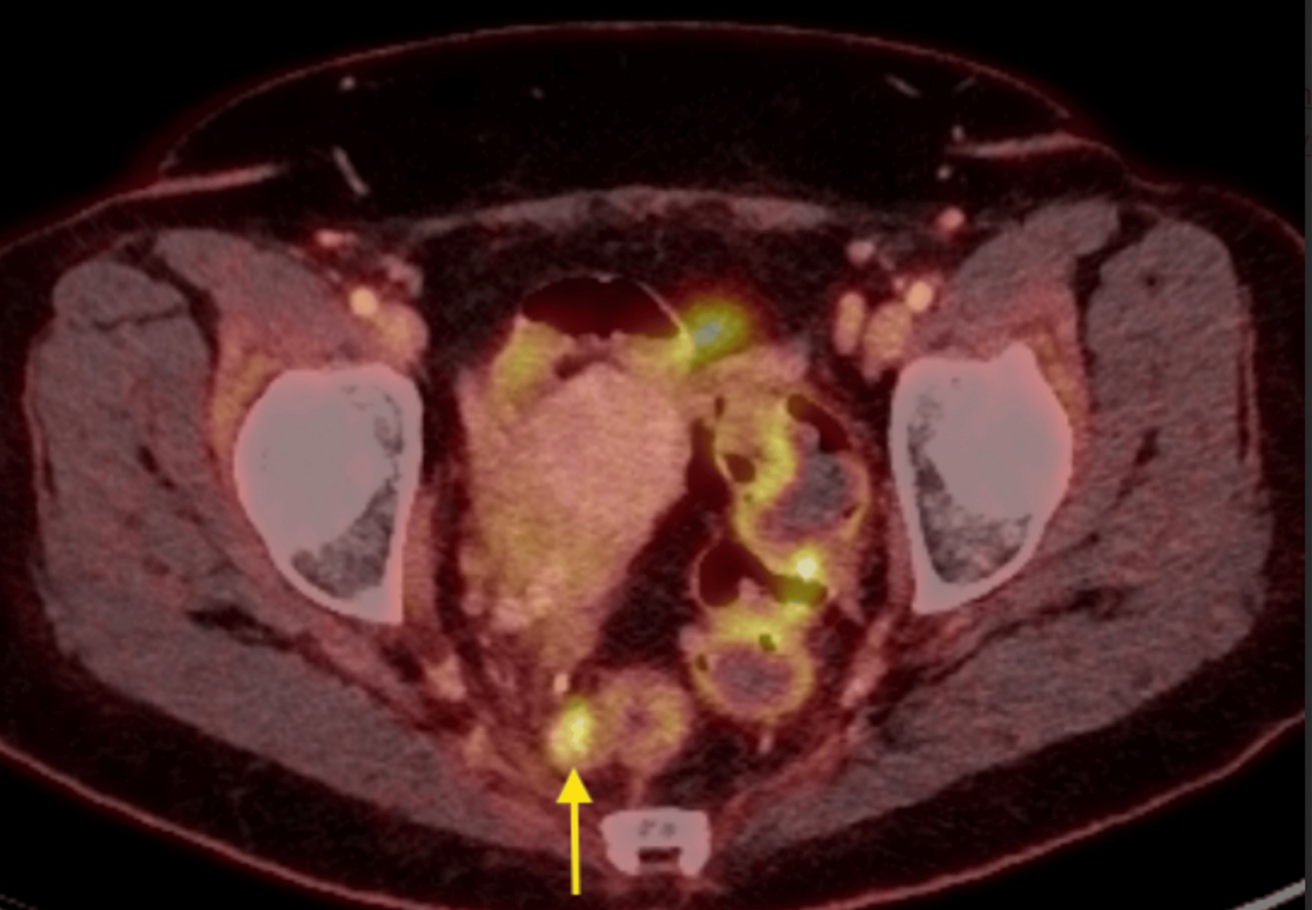
Axial fused PET/CT image demonstrating a right perirectal mass (yellow arrow) that is stable in size and has SUV=6. PET/CT: positron emission tomography/computed tomography; SUV: standardized uptake value

The most recent MRI shows an overall decreased presacral lesion size, now measuring 1.5 x 1.6 cm (Figures 10, 11). The patient was presented again at a multidisciplinary tumor board, including colorectal surgery. In the discussion, given that the tumor appeared to separate and pull away from the cervix and the residual disease is pararectal, the tumor was reclassified as an atypical presentation of anal SCC. Colorectal surgery assumed care for the management of the residual disease, and the patient underwent an uncomplicated abdominoperineal resection, end colostomy, and total hysterectomy with bilateral salpingo-oophorectomy. The final pathology on the resection specimen was negative for viable residual disease, and clinical staging was confirmed as stage IIIC rectal SCC.

## Discussion

An initial evaluation of a CUP includes a detailed history, physical exam, and tissue biopsy. The National Comprehensive Cancer Network (NCCN) Clinical Practice Guidelines in Oncology for Occult Primary suggest additional evaluation depending on the pathologic subset of CUP found. For SCC, this includes an abdominal/pelvic CT, detailed gynecologic and anal examination, consultation with gynecologic oncology, and anal endoscopy [1]. Although rare, SCC with inguinal lymph nodes is a favorable prognostic factor for detecting the primary site and undergoing treatment [8]. Our patient had positive pelvic nodes and negative inguinal nodes on imaging, making this a more challenging diagnostic question.

High-risk HPV, commonly types 16 and 18, has oncogenic potential for causing invasive disease in the oropharyngeal, gynecologic, and lower gastrointestinal sites. Although oropharyngeal cancer is the most common HPV-associated cancer overall, cervical cancer is the most common HPV-associated cancer in women [3]. The incidence of HPV-associated cervical cancer in women is approximately 7.6 per 100,000 in the United States and is decreasing secondary to screening and vaccination strategies [9]. On the other hand, the incidence of anal cancer is about two per 100,000 and, although rare, is on the rise [9,10].

One of the most frequently used IHC markers is p16, a surrogate marker for oncogenic HPV infection [4]. However, it is a non-specific marker and positive in non-HPV-related cancers such as Hodgkin and non-Hodgkin lymphomas, gastric and pulmonary adenocarcinomas, liposarcomas, neuroendocrine carcinomas, and a subgroup of uterine carcinomas [3]. The use of RNA ISH improves the sensitivity and specificity of the detection of HPV-related lesions. Thus, HPV RNA ISH provides the advantage of detection of active viral E6/E7 mRNA [11]. In our case, both p16 and RNA ISH for HPV were positive. This is expected with cervical cancer but is not specific to it, as we have discussed.

The backbone of treatment for CUP SCC is chemoradiation. The specific regimen depends on the primary site of origin. According to the NCCN guidelines, the standard of care for locally advanced cervical SCC consists of radiation with cisplatin, whereas the standard for anal SCC often combines mitomycin with 5-fluorouracil (5-FU) [1,12]. Our patient completed a course of cisplatin combined with VMAT radiation for presumed cervical primary with minimal response in tumor burden. Further response was achieved when the radiation approach changed to SBRT.

In our case, the primary site was presumed cervical in origin in the setting of a female patient with a p16-positive and HPV-positive tumor in the pelvis, HPV-negative anal biopsies, and a higher incidence of cervical cancer compared to anal cancer. Our initial treatment course was subject to our anchoring bias on the wrong primary cancer type based on the most likely diagnosis. While there was limited response with a cervical-based regimen, tumor response greatly improved after switching the treatment regimen to a standard treatment for anal cancer. Looking back on this case, using an anal cancer regimen earlier could have had several benefits. First, the dose and duration of chemoradiation the patient was subjected to was longer than necessary, given the switch in the treatment course, and this can have toxicities such as cystitis, vaginitis, vaginal ulceration or fistulas, and surrounding skin damage. Second, earlier identification of the correct diagnosis and treatment would have reduced healthcare costs by reducing clinic appointments and total treatment costs. In our case, however, the mass effect of the tumor on surrounding pelvic structures made the origin of the tumor difficult to initially assess until chemoradiation shrunk the mass.

Choosing the appropriate chemoradiation was key in decreasing the tumor burden in this patient. The data for adjuvant SBRT in lieu of brachytherapy is limited [7,12-16]. However, by limiting the OARs of the rectum, sigmoid, bowel, and bladder and calculating doses to the planning target volume with a goal of 80-95 Gy, many patients were able to be treated in three to five fractions given twice weekly, comparable to brachytherapy fractionation [17,18]. As there is no firm standard for SBRT fractionation in this setting, this fractionation was chosen to best provide a definitive dose to the primary anal tumor while also attempting to spare OARs.

Multidisciplinary teams are essential to help reduce the risk of anchoring bias during the diagnosis and treatment of CUP. Anchoring effects and other cognitive biases are known to contribute to diagnostic inaccuracies [19]. Furthermore, anchoring bias can be reduced by interventions that provide "cognitive help," including consulting with other specialists [20]. The primary team can be anchored by the initial presentation and data, but the additional perspectives of other specialties can expand the differential diagnosis and allow for uncoupling from this bias.

## Conclusions

CUP are rare malignancies that are challenging to treat. Detection of HPV-related lesions can be enhanced by using immunohistochemistry markers such as p16 combined with RNA ISH. Still, the diagnosis of the primary tumor may be elusive; therefore, when treating a CUP, providers should maintain a high degree of suspicion to reevaluate treatment regimens when the expected tumor response is less than expected. Multidisciplinary teams are essential for addressing anchoring bias and providing the best patient outcomes.
